# Assessment of a cellular host response test to risk-stratify suspected COVID-19 patients in the Emergency Department setting

**DOI:** 10.1371/journal.pone.0264220

**Published:** 2022-03-16

**Authors:** Hollis R. O’Neal, Roya Sheybani, Terrell S. Caffery, Diana Hamer, L. Mark Laperouse, Mandi W. Musso, Catherine S. O’Neal, Henry T. K. Tse, Ajay M. Shah, Christopher B. Thomas

**Affiliations:** 1 Louisiana State University Health Sciences Center, Baton Rouge, Louisiana, United States of America; 2 Our Lady of the Lake Regional Medical Center, Baton Rouge, Louisiana, United States of America; 3 Cytovale, Inc., San Francisco, California, United States of America; National Institute of Child Health and Human Development (NICHD), NIH, UNITED STATES

## Abstract

**Objective:**

Assess the IntelliSep Index (ISI) for risk stratification of patients presenting to the Emergency Department (ED) with respiratory symptoms suspected of COVID-19 during the pandemic.

**Methods:**

An observational single-center study of prospective cohort of patients presenting to the ED during the early COVID-19 pandemic with respiratory symptoms and a CBC drawn within 4.5 hours of initial vital signs. A sample of this blood was aliquoted for performance of the ISI, and patients were followed for clinical outcomes. The study required no patient-centered activity beyond standard of care and treating clinicians were unaware of study enrollment and ISI test results.

**Main findings:**

282 patients were included. The ISI ranges 0.1 to 10.0, with three interpretation bands indicating risk of adverse outcome: low (green), 0.1–4.9; intermediate (yellow), 5.0–6.2; and high (red), 6.3–10.0. Of 193 (68.4%) tested for SARS-CoV-2, 96 (49.7%) were positive. The ISI resulted in 182 (64.5%) green, 54 (18.1%) yellow, and 46 (15.6%) red band patients. Green band patients had a 1.1% (n = 2) 3-day mortality, while yellow and red band had 3.7% (n = 2, p > .05) and 10.9% (n = 5, p < .05) 3-day mortalities, respectively. Fewer green band patients required admission (96 [52.7%]) vs yellow (44 [81.5%]) and red (43 [93.5%]). Green band patients had more hospital free days (median 23 (Q1-Q3 20–25) than yellow (median 22 [Q1-Q3 0–23], p < 0.05) and red (median 21 [Q1-Q3 0–24], p < 0.01). SOFA increased with interpretation band: green (2, [Q1-Q3 0–4]) vs yellow (4, [Q1-Q3 2–5], p < 0.001) and red (5, [Q1-Q3 3–6]) p < 0.001).

**Conclusions:**

The ISI rapidly risk-stratifies patients presenting to the ED during the early COVID-19 pandemic with signs or suspicion of respiratory infection.

## Introduction

Emerging and re-emerging pathogens are a persistent threat to the public health [1]; indeed, the COVID-19 pandemic, caused by worldwide circulation of severe acute respiratory syndrome coronavirus-2 (SARS-CoV-2), has illustrated this threat. As a result of complex host-pathogen interactions [2], infection with SARS-CoV-2 results in a spectrum of clinical disease with widely variable severity [3–7], from asymptomatic carriage to a dysregulated immune response that leads to organ dysfunction and death [8–11]. No specific clinical features discern COVID-19 from other respiratory illnesses [12]. Throughout this pandemic, as hospitals filled with patients under investigation for COVID-19, clinicians attempted to discern high-risk patients at who need aggressive intervention from lower-risk patients who may benefit from a more conservative approach [13].

Timely recognition of patients with a dysregulated immune response may facilitate decision making and optimize both clinical care and resource utilization [14] in situations where the pathogen is poorly understood or when rapid diagnostic and pathogen identification tests are not available. In these situations, a pathogen-agnostic assessment of the host response may assist clinicians in the risk stratification of persons under investigation for infection by providing a patient-centered, objective assessment of the state of immune activity.

By assessing the biophysical properties of thousands of leukocytes [15, 16], we are able to quantify the state of innate immune activation in the Intellisep Index (ISI) [17], which we have shown to have the potential to be a rapid, reliable diagnostic for sepsis in patients presenting to the emergency department with signs or suspicion of infection [18]. To explore the utility of the ISI in the setting of a pandemic caused by a novel pathogen, we conducted a prospective, observational study of patients presenting to the ED of a large academic medical center with respiratory symptoms early in the COVID-19. Here, we report the performance of this tool as a means to stratify this population for risk of morbidity—especially respiratory—and mortality early in the course of the ED visit, independent of the patient’s SARS-CoV-2 infection status.

## Methods

This is an observational single-center, prospective cohort that required no patient-centered activity beyond standard of care. The ISI results were experimental only, and treating clinicians were aware of neither study enrollment nor test results. The Louisiana State University Health Sciences Center Institutional Review Board approved the study protocol and granted waivers of informed consent (Louisiana State University Health Sciences Center–New Orleans, Human Subjects Research Protection Program and Institutional Review Board: LSUHSC-NO #20–048; ClinicalTrials.gov NCT04372472).

### Study population and sample collection

During the study period of April 07 through April 28, 2020 Louisiana’s rate of incident SARS-CoV-2 infection was among the highest in the U.S. [19, 20]. We screened 307 adult patients who presented to the ED’s respiratory care unit of Our Lady of the Lake Regional Medical Center, an 800-bed academic medical center in Baton Rouge, Louisiana. Only patients with a complete blood count (CBC) collected were included in the study. Research staff aliquoted 0.3 to 1.0 milliliters of whole blood from the EDTA-anticoagulated tube (drawn for the CBC) for ISI measurement. The protocol excluded those with an insufficient volume of blood or invalid ISI results. Laboratory technicians completed the test within five hours of venipuncture to avoid possible sample degradation.

### IntelliSep test

The IntelliSep test (Cytovale Inc., San Francisco, CA) is an investigational laboratory test conducted on 100 microliter sample of whole blood [17]. It assesses intracellular and nuclear changes that occur during leukocyte activation by measuring the biophysical parameters of tens of thousands of leukocytes in less than 10 minutes. The test provides a single score, the IntelliSep Index (ISI), between 0.1–10.0, (inclusive), stratified into three discrete interpretation bands of risk for morbidity and mortality: green (low-risk, 0.1–4.9), yellow (intermediate-risk, 5.0–6.2), and red (high-risk, 6.3–10.0). The green band includes the 95% Confidence Interval of measurements from healthy donors. The algorithm for calculating the ISI and limits for interpretation bands were defined based on all-cause sepsis-focused studies prior to March 2020 [17]. No information from patients in this study influenced the calculations or interpretation band of the ISI.

### Data collection

Study coordinators collected demographic information, baseline data, comorbidities, physiologic data, laboratory values, and need for life support through chart review. Coordinators collected radiographic, historical, physical and laboratory data from the medical record for the identification of specific infections based on pre-defined criteria. In addition, coordinators calculated baseline Sequential Organ Failure Assessment (SOFA) scores [21] and daily SOFA scores for each of the first three days of hospitalization. Other than the ISI, available data were a result of standard care. Details for the calculation of SOFA scores is provided in the S1 File; criteria for assignment of objective infection are modified from the Centers for Disease Control and Prevention’s National Healthcare Safety Network criteria [22]. Calculation of APACHE II is described elsewhere [23].

### Statistical analysis

Unless otherwise stated, p-values were obtained from an unpaired two-sample Welch’s t-test or Mann-Whitney U, as appropriate, where the null hypothesis is that the mean of the two samples are equal. Descriptive statistics are presented as means, standard deviations, medians, and interquartile ranges for the continuous variables, and as counts and percentages for categorical variables. An alpha level of 5% is used for all analyses, unless otherwise stated. Two-sided confidence intervals for proportions are provided using the Clopper-Pearson method, where appropriate. Time-to-event analyses are depicted with Kaplan-Meier plots, and the log-rank test was used for comparison between groups.

## Results

**Fig 1:**
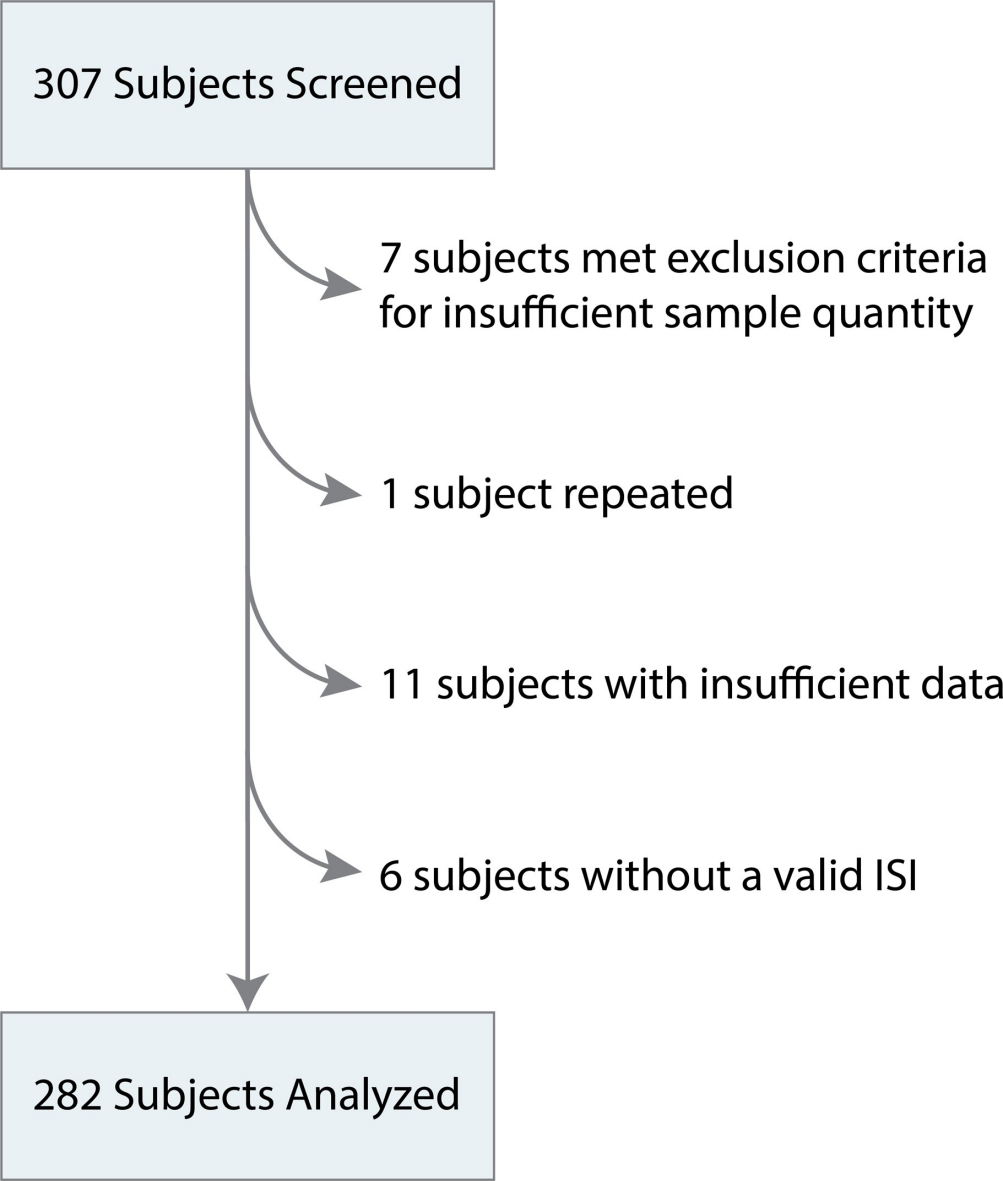
307 adult patients with suspicion of respiratory infection for which a complete blood count was ordered in the emergency department were screened, 7 subjects met exclusion criteria for insufficient sample quantity and were not enrolled, 1 subject was enrolled repeatedly, and the second enrollment was excluded. Due to user error, test data was not recorded for 10 subjects and for 6 subject samples, testing did not produce a valid ISI. As a result, 282 subjects were included in the study analysis.

Table 1 contains baseline characteristics and comparative statistical descriptions for the overall study population and the subsets of patients in the green, yellow and red ISI bands. Coordinators screened 307 eligible patients for the study, and 282 patients had sufficient volume of blood sample and evaluable data to be included in the study (Fig 1). The median age was 61 years, 138 (49%) were men, 114 (40%) were white, 148 (52%) were black, and 20 (7%) were members of other races. In total, 149 patients (53%) met at least one objective infection criteria, including 110 (39%) respiratory, 30 (11%) urinary, and 20 (7%) gastrointestinal infections. Comorbidities included 172 (61%) hypertension, 81 (29%) diabetes, 105 (37%) obesity, and 33 (12%) cancer.

**Table 1:** Baseline characteristics and selected outcomes of all evaluable patients (total) as well as stratified by ISI interpretation band.

Characteristic		Total N = 282	Green Band N = 182	Yellow Band N = 54	Red Band N = 46	p-value
Age, median (Q1-Q3)		60.5 (46–73)	56.5 (46–73) *, ⱡ	65 (51.5–76) ⱡ	64.5 (49–76) *	* p < 0.05; ⱡ p < 0.01
Gender (Female), N (%)		144 (51.1)	98 (53.8)	24 (44.4)	22 (47.8)	NS
Race, N (%)	White	114 (40.2)	68 (37.7) *	21 (38.9)	25 (54.3) *	* p < 0.05
	African American	148 (52.5)	101 (55.5) *	30 (55.5)	17 (37.0) *	
	Other	20 (7.1)	13 (7.1)	3 (5.6)	4 (8.7)	
Comorbidities (N, %)	Hypertension	172 (61)	106 (58)	35 (65)	31 (67)	NS
	Diabetes	81 (28.7)	46 (25.3)	16 (29.6)	19 (41.3)	
	Obesity (BMI > = 30)	105 (37.2)	63 (34.6)	20 (37.0)	22 (47.8)	
	Cancer	33 (11.7)	21 (11.5)	7 (13.0)	5 (10.9)	
Infected—Meeting Objective Criteria (N, %), by organ system:	Respiratory	110 (39.0)	51 (28.0) *, ⱡ	24 (44.4) *, ⱡⱡ	35 (76.1) ⱡ, ⱡⱡ	* p < 0.05; ⱡ p < 0.001; ⱡⱡ p < 0.01
	Gastrointestinal	20 (7.1)	16 (8.8)	2 (3.7)	2 (4.3)	NS
	Urinary	30 (10.6)	13 (7.1) *	6 (11.1)	11 (23.9) *	* p < 0.05
	Other	14 (5.0)	3 (1.6) *	5 (9.2)	6 (13.0) *	* p < 0.05
	Not Infected	133 (47.2)	107 (58.8) *, ⱡ	22 (40.7) *, ⱡⱡ	4 (8.7) ⱡ, ⱡⱡ	* p < 0.05; ⱡ p < 0.001; ⱡⱡ p < 0.001
SARS-CoV-2 Testing (N, %)	Number Tested	193 (68.4)	104 (57.1) ⱡ, ⱡⱡ	49 (90.7) ⱡ	40 (87.0) ⱡⱡ	ⱡ p < 0.001; ⱡⱡ p < 0.001
	Number Positive (of tested)	96 (49.7)	53 (51.0)	23 (46.9)	20 (50.0)	NS
	Number Positive (of total)	96 (34.0)	53 (29.1)	23 (42.6)	20 (43.4)	NS
Blood Culture (N, %)	Number Tested	114 (40.4)	51 (28.0) ⱡ, ⱡⱡ	30 (55.6) ⱡ	33 (71.7) ⱡⱡ	ⱡ p < 0.001; ⱡⱡ p < 0.001
	Number Positive (of tested)	14 (12.3)	3 (5.9)	4 (13.3)	7 (21.2)	NS
Admitted to Hospital (N, %)		183 (64.9)	96 (52.7) ⱡ, ⱡⱡ	44 (81.5) ⱡ	43 (93.5) ⱡⱡ	ⱡ p < 0.001; ⱡⱡ p < 0.001
Admitted to ICU (N, %)		52 (18.4)	24 (13.2) ⱡ	10 (18.5) *	18 (39.1) ⱡ, *	ⱡ p < 0.01; * p < 0.05
Hospital Free Days (Admitted Subjects), median (Q1-Q3)		22 (11.5–24)	23 (20–25) ⱡ, *	21.5 (0–23) *	21 (0–23.5) ⱡ	ⱡ p < 0.01; * p < 0.05
Mortality, N (%)	3-day	9 (3.2)	2 (1.1) *	2 (3.7)	5 (10.9) *	* p < 0.05
	7-day	18 (6.4)	3 (1.6) *, ⱡ	6 (11.1) *	9 (19.6) ⱡ	* p < 0.05; ⱡ p < 0.01
	In-hospital	40 (14.2)	14 (7.7) *, ⱡ	12 (22.2) *	14 (30.4) ⱡ	* p < 0.05; ⱡ p < 0.01
SOFA, 3-day max, median (Q1-Q3)		3 (1–5)	2 (0–4) ⱡ, ⱡⱡ	4 (2–5) ⱡ	4.5 (3–6) ⱡⱡ	ⱡ p < 0.001; ⱡⱡ p < 0.001
Maximum SOFA change from enrollment for those admitted, median (Q1-Q3)		0 (-1-1)	0 (-1-1)	0 (-1-2)	0 (-2-1)	NS
APACHE II, median (Q1-Q3)		11 (6–17)	9 (4–13) ⱡ, ⱡⱡ	14 (9–18) ⱡⱡ	14 (11–19) ⱡ	ⱡ p < 0.001; ⱡⱡ p < 0.001
P/F ratio, 3-day min, median (Q1-Q3)		295 (189–414)	329 (221–457) ⱡ, ⱡⱡ	262 (158–321) ⱡ	218 (151–304) ⱡⱡ	ⱡ p < 0.001; ⱡⱡ p < 0.001
WBC (10^3^ cells/μL), median (Q1-Q3)		8.2 (5.9–11.9)	7.3 (5.6–10.3) ⱡⱡ	9.6 (7.0–12.7) ⱡⱡ, ⱡ	11.9 (7.0–18.0) ⱡ	ⱡⱡ p < 0.01; ⱡ p < 0.001
Segmented Neutrophils (%), median (Q1-Q3)		74 (64–82)	69 (59–77) ⱡ, ⱡⱡ	80 (78–86) ⱡ	82 (76–88) ⱡⱡ	ⱡ p < 0.001; ⱡⱡ p < 0.001
Lymphocytes (%), median (Q1-Q3)		17 (10–26)	23 (16–30) ⱡ, ⱡⱡ	11 (7–15) ⱡ	8 (6–13) ⱡⱡ	ⱡ p < 0.001; ⱡⱡ p < 0.001
Monocytes (%), median (Q1-Q3)		6 (4–9)	6 (5–9)	6 (4–9)	6 (4–8)	NS
Platelets (10^3^ cells/μL), median (Q1-Q3)		230 (172–294)	229 (175–288)	223 (159–271)	243 (163–330)	NS
Blood Urea Nitrogen (mg/dl), median (Q1-Q3)		16 (11–28)	14 (10–22) ⱡ	28 (14–52) *, ⱡ	20 (14–33) *	* p < 0.05; ⱡ p < 0.001
Creatinine(mg/dl), median (Q1-Q3)		1.10 (0.81–1.55)	1.01 (0.79–1.32)	1.53 (1.06–2.86)	1.13 (0.96–1.99)	NS
Triage Temperature (F), median (Q1-Q3)		98.7 (98.0–99.7)	98.5 (98.0–99.3) ⱡ	99.2 (98.0–100.1) ⱡ	99.1 (98.0–101.0)	ⱡ p < 0.01
IntelliSep Index, median (Q1-Q3)		4.2 (3.2–5.6)	3.4 (2.6–4.1) *, ⱡ	5.6 (5.2–5.9) ⱡ, ⱡⱡ	7.3 (6.7–8.0) *, ⱡⱡ	* p < 0.001; ⱡ p < 0.001; ⱡⱡ p < 0.001

**Abbreviations:** Q1-Q3, interquartile range. N, number. BMI, body mass index. NS, not significant.

p-values were obtained from an unpaired two-sample Welch’s t-test (except for hospital free days, where the Mann–Whitney U was due to the non-normal distribution), with the null hypothesis that the mean of the two samples are equal.

Of the 282 patients in the overall study population, clinicians tested 193 (68%) for SARS-CoV-2 infection shortly prior to or during the ED encounter or associated admission, and 96 (34% of total, 50% of tested) were positive. In the majority of cases, the ED clinicians were unaware of SARS-CoV-2 infection status, as molecular diagnostics at the time of enrollment resulted at least 24 hours after collection (a minority of patients tested positive prior to the ED counter associated with enrollment). A total of 183 (65%) patients were admitted to the hospital with a median length of stay of 5 days, and 52 (18%) were admitted to the Intensive Care Unit (ICU). Forty (14% of total, 22% of admitted) patients died while in the hospital.

The median baseline SOFA score of all patients was 0 (Q1-Q3, 0–1). The median SOFA score for all patients was 2 on Day 1 (Q1-Q3, 1–4), 3 on Day 2 (Q1-Q3, 2–5), and 2 on Day 3 (Q1-Q3, 2–4). The median APACHE-II score for all patients, calculated from the worst values in the date of the ED encounter, was 11 (Q1-Q3, 6–17).

The median ISI for all patients was 4.2. Using prespecified cutoffs for ISI, 182 (64%) were classified as green, 54 (19%) yellow, and 46 (16%) red. The red band had a median age of 64.5 years and included a higher percentage of white patients (54.3%). By comparison, the green band had a median age of 56.5 (p < .05) and included a higher percentage of black patients (55.5%, p < 0.05). The red band also had higher percentages of patients who were diabetic (41.3%) or obese (47.8%), as compared to the green (25.3% diabetic, p > 0.05 and 34.6% obese, p > 0.05).

Patients with SARS-CoV-2 infection had baseline demographics similar to the overall study population, both in total and when stratified by ISI risk category (Table 2). Of the 96 patients who tested positive for SARS-CoV-2 infection, 53 (55%) were classified into the green band, 23 (24%) yellow, and 20 (21%) red.

**Table 2:** Baseline characteristics and selected outcomes of SARS-CoV-2 positive patients (total) as well as stratified by ISI interpretation band.

Characteristic		Total N = 96	Green Band N = 53	Yellow Band N = 23	Red Band N = 20	p-value
Age, median (Q1-Q3)		65 (51–75)	61 (47–73)	65 (52–69)	70 (58–80)	NS
Gender, N (%)	Female	46 (47.9)	29 (54.7)	8 (34.8)	9 (45.0)	NS
	Male	50 (52.1)	24 (45.3)	15 (65.2)	11 (55.0)	
Race, N (%)	White	30 (31.3)	15 (28.3) *	4 (17.4) ⱡ	11 (55.0) *, ⱡ	* p < 0.05
	African American	57 (59.4)	32 (60.4)	18 (78.3) ⱡⱡ	7 (35.0) ⱡⱡ	ⱡ p < 0.05
	Other	9 (9.4)	6 (11.3)	1 (4.3)	2 (10.0)	ⱡⱡ p < 0.01
Comorbidities (N, %)	Hypertension	67 (69.8)	33 (62.3)	18 (78.3)	16 (80.0)	NS
	Diabetes	38 (42.2)	17 (36.2)	10 (43.5)	11 (55.0)	
	Obesity (BMI 30–39.9)	33 (34.4)	20 (37.7)	6 (26.1)	7 (35.0)	
	Obesity (BMI 40–49.9)	7 (7.3)	3 (5.7)	2 (8.7)	2 (10.0)	
	Obesity (BMI > = 50)	2 (2.1)	1 (1.9)	0 (0.0)	1 (5.0)	
	Cancer	7 (7.3)	4 (7.5)	2 (8.7)	1 (5.0)	
Infected—Meeting Objective Criteria (N, %), by organ system:	Respiratory	63 (65.6)	31 (58.5) *	15 (65.2)	17 (85.0) *	* p < 0.05
	Gastrointestinal	3 (3.1)	3 (5.7)	0 (0.0)	0 (0.0)	
	Urinary	9 (9.4)	3 (5.7)	1 (4.3)	5 (25.0)	
	Other	1 (1.0)	1 (1.9)	0 (0.0)	0 (0.0)	
	Not Infected	27 (28.1)	17 (32.1) *	8 (34.8)	2 (10.0) *	
Blood Culture (N, %)	Number Tested	46 (47.9)	21 (39.6) *	11 (47.8)	14 (70.0) *	* p < 0.05
	Number Positive (of tested)	1 (2.2)	1 (4.8)	0 (0.0)	0 (0.0)	NS
Admitted to Hospital (N, %)		79 (82.3)	40 (75.5) ⱡⱡ	19 (82.6) ⱡ	20 (100.0) ⱡⱡ, ⱡ	ⱡ p < 0.05
						ⱡⱡ p < 0.001
Admitted to ICU (N, %)		20 (20.8)	8 (15.1)	4 (17.4)	8 (40.0)	NS
Hospital Free Days (Admitted Subjects), median (Q1-Q3)		21 (0–24)	21 (10–24)	23 (6–24)	21 (0–23)	NS
Mortality, N (%)	3-day	4 (4.2)	0 (0.0)	1 (4.3)	3 (15.0)	NS
	7-day	10 (10.4)	1 (1.9) *	2 (8.7) ⱡ	7 (35.0) *, ⱡ	* p < 0.01
						ⱡ p < 0.05
	In-hospital	24 (25.0)	9 (17.0) *	6 (26.1)	9 (45.0) *	* p < 0.05
SOFA, 3-day max, median (Q1-Q3)		3 (2–5)	3 (1–4) *	4 (3–5) *	3 (2–7)	* p < 0.05
Maximum SOFA change from enrollment for those admitted, median (Q1-Q3)		0 (-1-1)	-1 (-2-1)	-1 (-2-1)	0 (-1-1)	NS
APACHE II, median (Q1-Q3)		13 (7–17)	11 (4–14) ⱡ, ⱡⱡ	15 (11–18) ⱡⱡ	17 (13–20) ⱡ	ⱡ p < 0.001
						ⱡⱡ p < 0.01
P/F ratio, 3-day min, median (Q1-Q3)		246 (152–329)	261 (164–329)	243 (155–303)	183 (114–310)	NS
WBC (10^3^ cells/μL), median (Q1-Q3)		7.0 (5.4–9.5)	6.0 (5.2–7.9) ⱡ	7.8 (6.6–10.3)	7.2 (6.0–13.8) ⱡ	ⱡ p < 0.05
Segmented Neutrophils (%), median (Q1-Q3)		74 (67–80)	69 (58–77) *, ⱡ	79 (73–82) *	80 (74–85) ⱡ	* p < 0.001
						ⱡ p < 0.05
Lymphocytes (%), median (Q1-Q3)		17 (11–24)	23 (15–31) ⱡ, ⱡⱡ	12 (10–17) ⱡ	12 (7–15) ⱡⱡ	ⱡ p < 0.001
						ⱡⱡ p < 0.001
Monocytes (%), median (Q1-Q3)		6 (4–9)	6 (4–8)	6 (4–9)	6 (4–8)	NS
Platelets (10^3^ cells/μL), median (Q1-Q3)		191 (152–256)	197 (147–256)	185 (154–243)	189 (154–261)	NS
Blood Urea Nitrogen (mg/dl), median (Q1-Q3)		21 (13–35)	18 (11–26) *	30 (15–49) *	26 (14–34)	* p < 0.05
Creatinine(mg/dl), median (Q1-Q3)		1.2 (0.8–1.9)	1.1 (0.8–1.4) *	1.8 (1.3–2.9) *	1.2 (0.9–2.1)	* p < 0.05
Triage Temperature, median (Q1-Q3)		99.1 (98.5–100.2)	99.1 (98.5–100.0)	99.5 (98.7–100.5)	99.0 (98.0–100.1)	NS
IntelliSep Index, median (Q1-Q3)		4.7 (3.7–5.9)	3.9 (3.3–4.4) *, ⱡ	5.4 (5.1–5.8) *, ⱡⱡ	7.1 (6.7–7.7) ⱡ, ⱡⱡ	* p < 0.001
						ⱡ p < 0.001
						ⱡⱡ p < 0.001

**Abbreviations:** Q1-Q3, interquartile range. N, number. BMI, body mass index. NS, not significant.

p-values were obtained from an unpaired two-sample Welch’s t-test (except for hospital free days, where the Mann–Whitney U was due to the non-normal distribution), with the null hypothesis that the mean of the two samples are equal.

### Mortality

**Fig 2:**
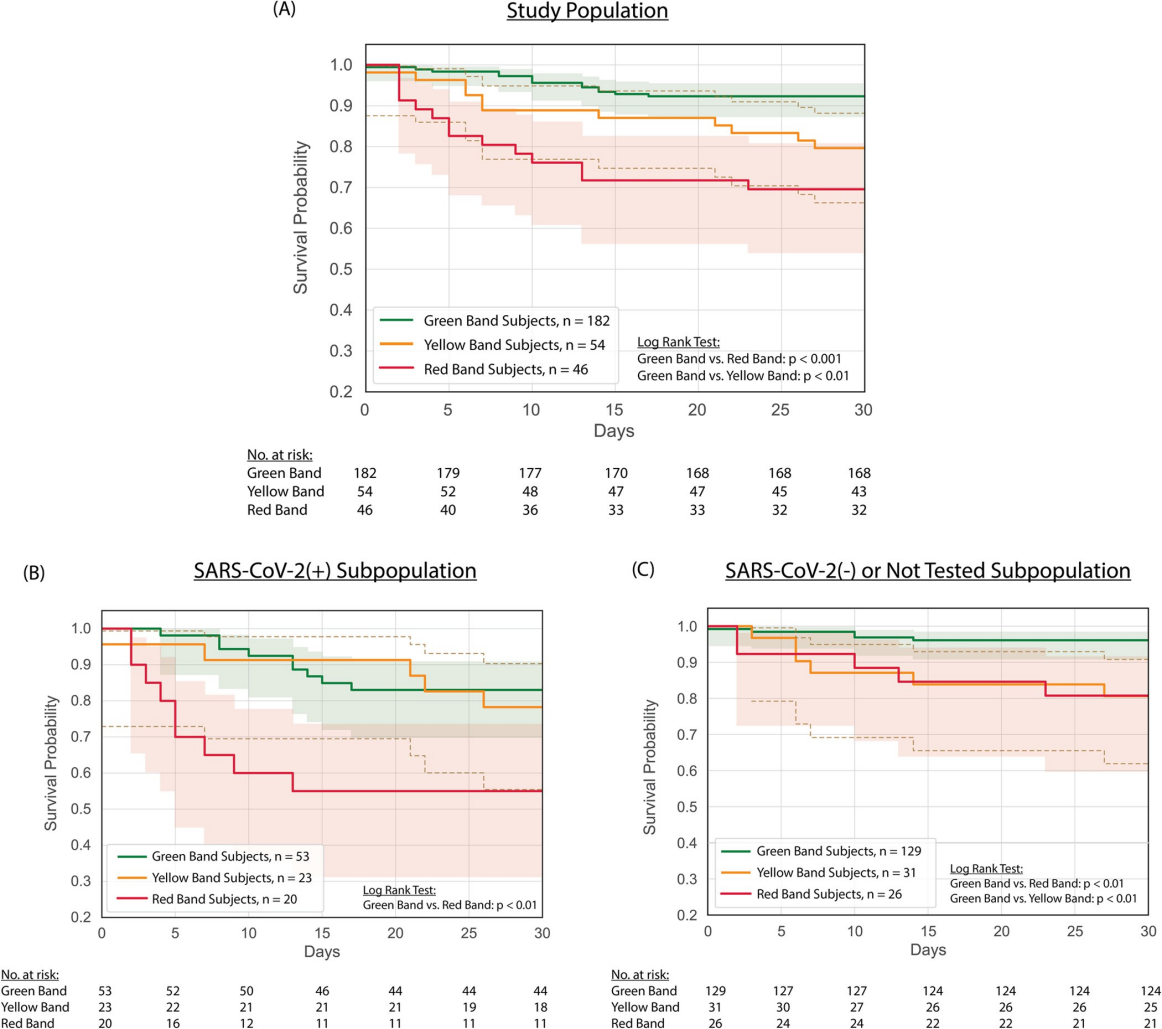
Kaplan-Meier survival stratified by ISI interpretation band for A) all evaluable subjects, B) subjects positive for SARS-CoV-2 infection by nucleic acid amplification test, and C) subjects that were either tested negative or not tested for SARS-CoV-2 infection. Plots were created with right-censoring, with the assumption that subjects discharged from the ED or hospital survived ≥ 30 days in the absence of evidence to the contrary (e.g., return to the ED, discharge to hospice, or other indication in the electronic health record, which was reviewed after 30 days, that the subject died). Shading depicts 95% confidence intervals. At each timepoint, the number at risk, per interpretation band, are noted below the figure panel.

Fig 2 depicts Kaplan-Meier survival curves stratified by ISI risk group. Among the 282 patients in the overall study population, the green band (n = 182) had a 3-day mortality of 1.1% and 7-day mortality of 1.6%, while the yellow band (n = 54) had a 3-day mortality of 3.7% and 7-day mortality of 11.1%, and the red band (n = 46) had a 3-day mortality of 10.9% and 7-day mortality of 19.6% (Fig 2A). Among the 96 patients that tested positive for SARS-CoV-2, those classified as green (n = 53) had a 3-day mortality of 0.0% and 7-day mortality of 1.9%, while patients classified as yellow (n = 23) had a 3-day mortality of 4.3% and 7-day mortality of 8.7%, and patients classified as red (n = 20) had a 3-day mortality of 15.0% and 7-day mortality of 35.0% (Fig 2B). These survival trends continue, with only 55% of those in the red band surviving at 15 days, compared to 87% of those in the green band. Of the remaining 186 patients, who were either not tested or were negative for SARS-CoV-2, 129 were in the green band with a 3-day mortality of 1.6% and 7-day mortality of 1.6%; 31 were in the yellow band with a 3-day mortality of 3.2% and 7-day mortality of 12.9%; 26 were in the red band with a 3-day mortality of 7.7% and 7-day mortality of 7.7% (Fig 2C).

### Severity of illness and utilization of hospital resources

**Fig 3:**
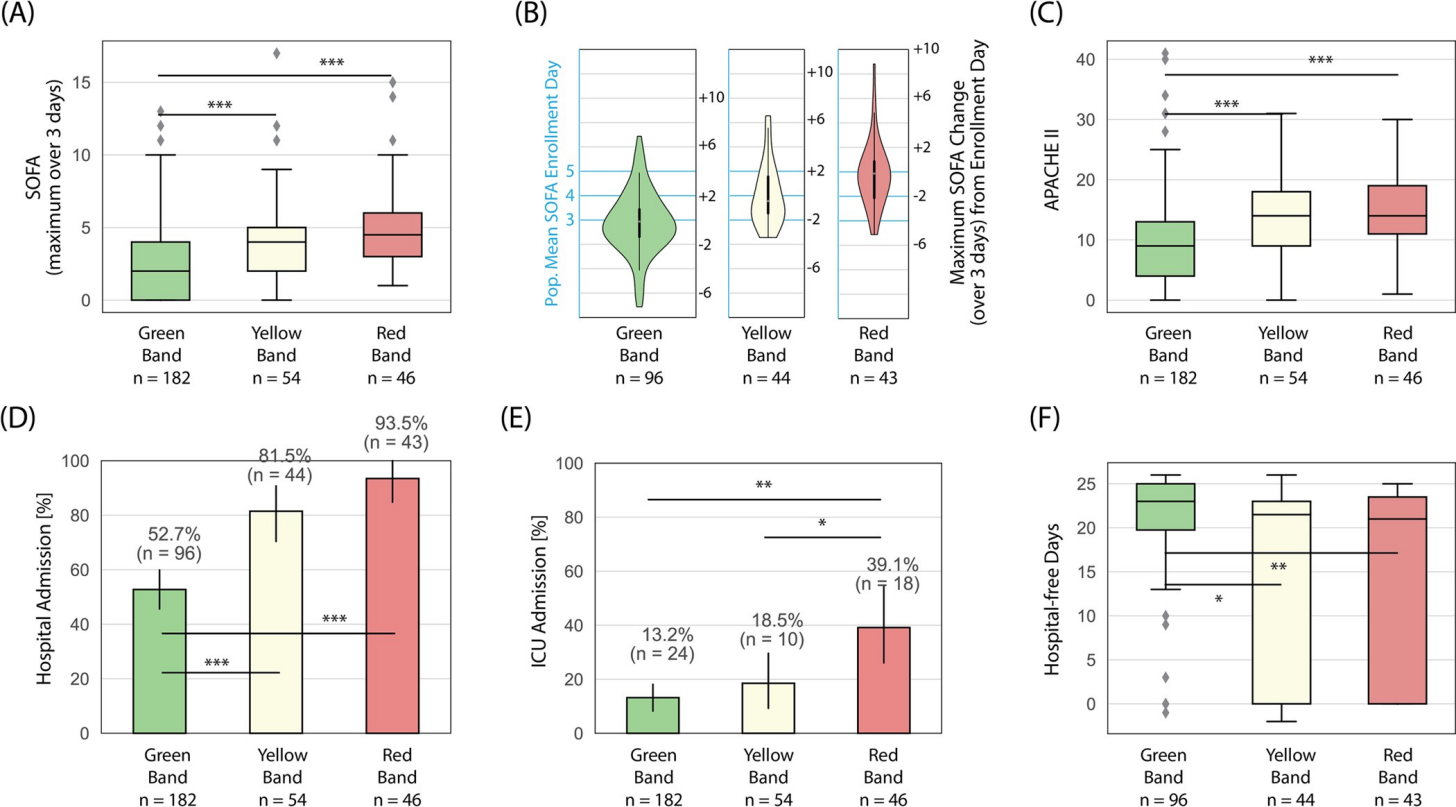
Trends in severity of illness scores and hospital utilization across ISI interpretation bands for all subjects. (A) SOFA scores, maximum calculated over up to 3-days with the subject’s baseline over the prior 6-months subtracted; (B) maximum SOFA change over 3-days from day of presentation; (C) APACHE II scores calculated using worse values over 24 hours following presentation; (D) hospital admission; (E) admission to the ICU; (F) hospital-free days in those admitted to the hospital, calculated as 28 –hospital length of stay, with zero for in-hospital mortality. Box plots: lines in the boxes, medians; the box ends, interquartile ranges (IQR); whiskers, 1.5x IQR; diamonds, outliers. Bar graphs: Bars, percentages; error pars, 95% confidence intervals. p-values were obtained from an unpaired two-sample Welch’s t-test (except for hospital free days, where the Mann–Whitney U was due to the non-normal distribution), with the null hypothesis that the mean of the two samples are equal. p-values reported as * p < 0.05, ** p < 0.01, and *** p < 0.001.

Fig 3 depicts severity of illness scores and hospital resource utilization for the overall study population, stratified by ISI group. ISI categories were consistent with severity of illness as measured by three-day maximum SOFA scores (Fig 3A). Patients in the red band had higher three-day maximum SOFA scores (median 5, Q1-Q3 = 3–6) than the green band (median 2, Q1-Q3 = 0–4) (p < 0.001). There was also a significant difference in three-day maximum SOFA scores between yellow (median 4, Q1-Q3 = 2–5) and green band patients (median 2, Q1-Q3 = 0–4) (p<0.001). There was no significant difference in three-day maximum SOFA scores between the red (median 5, Q1-Q3 = 3–6) and yellow patient groups (median 4, Q1-Q3 = 2–5) (p>0.05). Among admitted patients, those in the red band were more likely to have an increase in SOFA scores in second and third days of hospitalization compared to green band patients, who were more likely to have a decrease in SOFA scores during this time (Fig 3B).

ISI risk categories were also consistent with severity of illness as measured by APACHE-II (Fig 3C). Green band patients had a median APACHE-II score of 9 (Q1-Q3 = 4–13), while yellow band patients had a median score of 14 (Q1-Q3 = 9–18, p < 0.001), red band patients had a median score of 14 (Q1-Q3 = 11–19, p< 0.001). Additionally, red band patients were more likely to test positive for pathogens. Blood cultures were drawn for 33 red band patients, of which 7 (21% of tested) were positive, 30 yellow band patients of which 4 (13% of tested) were positive, and 51 green band patients of which 3 (6%) were positive (Table 1).

Differences in the utilization of hospital resources were also observed between interpretation bands. Hospital admissions were lowest for green band patients, with ED clinicians admitting 96 (53%) green band patients, 44 (82%) yellow band patients, and 43 (94%) red band patients (Fig 3D). Rates of admission to the intensive care unit (ICU) showed similar trends (Fig 3E), with red band patients more likely to require admission to the ICU (39%) compared to yellow band (19%, p < 0.05) and green band patients (13%, p< 0.01). Among those admitted to the hospital, green band patients had more hospital-free days (median 23, Q1-Q3 = 20–25) than yellow band (median 22, Q1-Q3 = 0–23) (p < 0.05) and red band patients (median 21, Q1-Q3 = 0–24) (p < 0.01) (Fig 3F).

**Fig 4:**
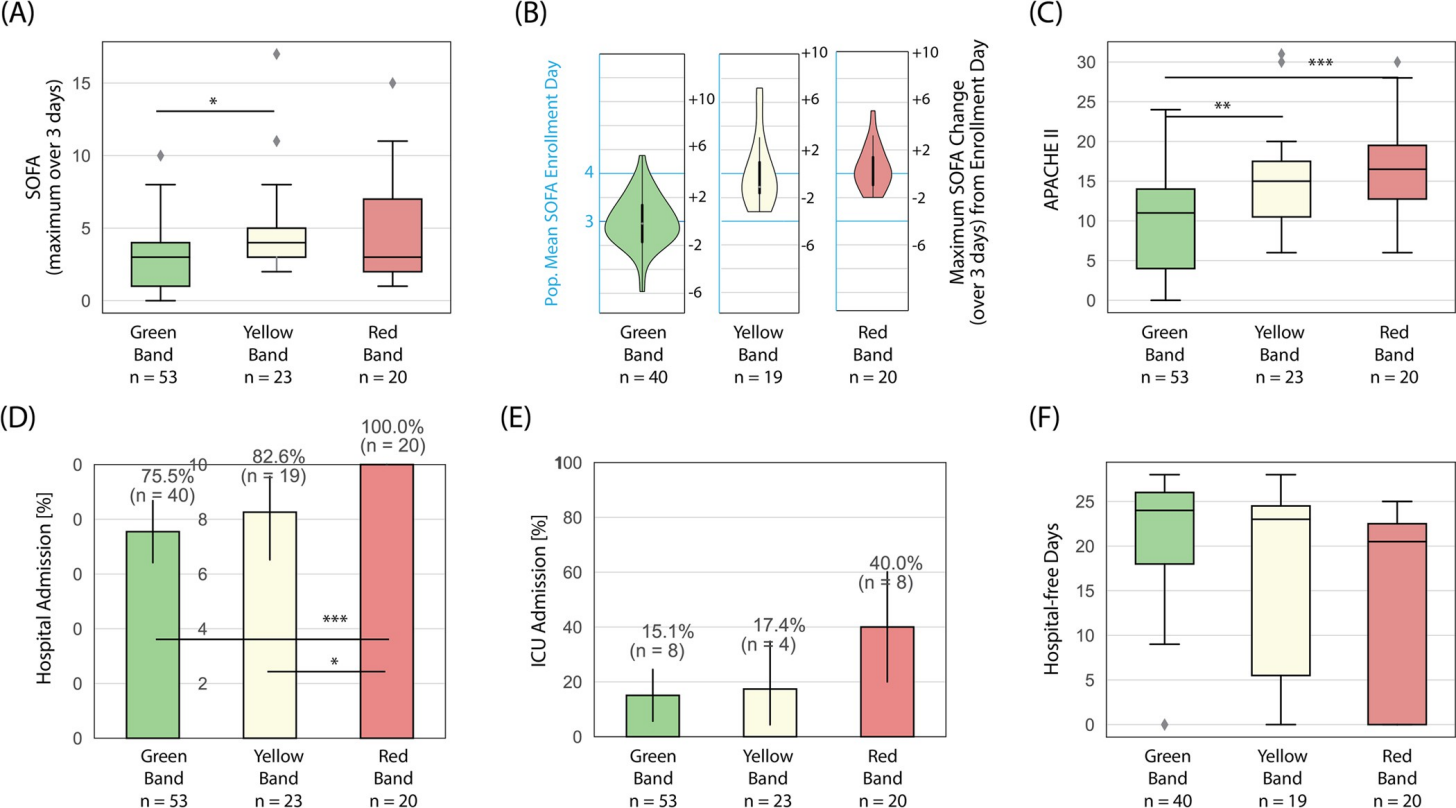
Trends in severity of illness scores and hospital utilization across ISI interpretation bands for SARS-CoV-2 positive subjects. (A) SOFA scores, maximum calculated over up to 3-days with the subject’s baseline over the prior 6-months subtracted; (B) maximum SOFA change over 3-days from day of presentation; (C) APACHE II scores calculated using worse values over 24 hours following presentation; (D) hospital admission; (E) admission to the ICU; (F) hospital-free days in those admitted to the hospital, calculated as 28 –hospital length of stay, with zero for in-hospital mortality. Box plots: lines in the boxes, medians; the box ends, interquartile ranges (IQR); whiskers, 1.5x IQR; diamonds, outliers. Bar graphs: Bars, percentages; error pars, 95% confidence intervals. p-values were obtained from an unpaired two-sample Welch’s t-test (except for hospital free days, where the Mann–Whitney U was due to the non-normal distribution), with the null hypothesis that the mean of the two samples are equal. p-values reported as * p < 0.05, ** p < 0.01, and *** p < 0.001.

Clinical trends observed for the ISI risk categories for the subset of patients that tested positive for SARS-CoV-2 (n = 96, 34% of study population) were consistent with the overall population (Fig 4). As compared to green band patients, red and yellow band patients had higher APACHE II and three-day maximum SOFA scores. Among those admitted to the hospital, red band and yellow band patients were more likely to have an increase in SOFA scores in second and third days of hospitalization. Red band patients also had higher rates of hospital and ICU admissions and, among those admitted to the hospital, fewer hospital-free days than those in the green band.

### Need for life support

**Fig 5:**
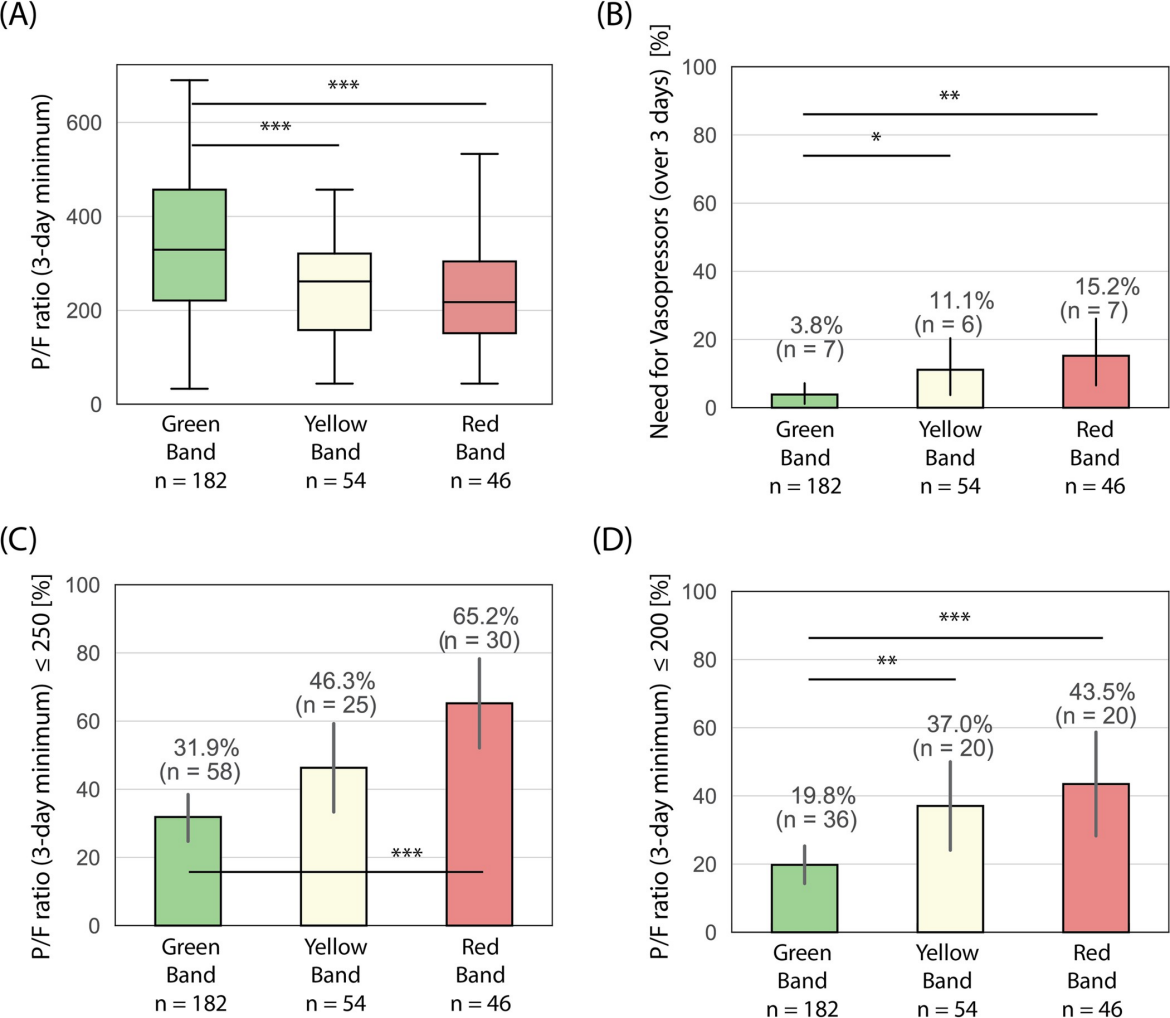
Trends in the need for life support (advanced oxygen-delivery and vasopressor support) across ISI interpretation bands for all subjects. (A) Minimum PaO2 / FiO2 ratio over up to 3-days, (B) need for vasopressors over up to 3-days; (C) minimum PaO2 / FiO2 ratio of less than 250 over up to 3-days; (D) minimum PaO2 / FiO2 ratio of less than 200 over up to 3-days. Box plots: lines in the boxes, medians; the box ends, interquartile ranges (IQR); whiskers, 1.5x IQR; diamonds, outliers. Bar graphs: Bars, percentages; error pars, 95% confidence intervals. p-values were obtained from an unpaired two-sample Welch’s t-test, with the null hypothesis that the mean of the two samples are equal. p-values reported as * p < 0.05, ** p < 0.01, and *** p < 0.001.

Patients with higher ISI scores were also more likely to require life support, including advanced oxygen-delivery and vasopressor support, in the first three days of hospitalization (Fig 5). Because of variability throughout the study period in the utilization of mechanical ventilators for SARS-CoV-2 positive patients, we defined the need for advanced oxygen-delivery as patients with a PaO_2_ / FiO_2_ ratio of less than 250. Additionally, to delineate a more severely-ill population, we also performed analysis for patients with a PaO_2_ / FiO_2_ ratio of less than 200. It is important to note, that in the calculation of PaO2 / FiO2 ratios, most FiO2 were estimated from flow rates on high-flow nasal cannula, with considerable variability between providers. Of patients in the green band, 32% had a minimum PaO_2_ / FiO_2_ ratio of less than 250 as opposed to 65% in the red band (p < 0.001). Additionally, 20% in the green band had a PaO_2_ / FiO_2_ ratio of less than 200 while 44% in the red band had a PaO_2_ / FiO_2_ ratio of less than 200 (p < 0.001). These observations were consistent in the entire study population as well as the population of patients who tested positive for SARS-CoV-2, albeit not statistically significant due to small sample size (S1 Fig in S1 File). Patients in the red band were also more likely to require vasopressor use than patients in the green band in both the entire population (Fig 5B) and in those testing positive for SARS-CoV-2 (S1B Fig in S1 File).

## Discussion

Patients often present to the ED with undifferentiated illnesses, forcing clinicians to make rapid, impactful decisions with limited data and prior to making the definitive diagnosis. As the early period of the COVID-19 pandemic demonstrated, the ED response is further complicated when the predominant pathogen is novel and poorly understood [6, 7, 24]. As with many infectious diseases, clinical risk factors and biomarkers were found to be associated with adverse outcomes in patients with COVID-19 [25–35]; however, there was no standardized process for interpreting these measures into clinically actionable results. As the pandemic progressed, COVID-19 specific scoring systems for predicting disease severity and mortality were developed, but they required numerous data inputs and observations for calculation which are not available early in the ED course [36–38]. An objective, standardized, rapid pre-diagnostic risk stratification system to aid ED clinicians in discerning patients in immediate jeopardy from those at lower risk could help maintain throughput without sacrificing quality of care in the setting of a novel pathogen [39]. Also, identification of patients with dysregulated immunity may improve allocation of resources, administration of specific interventions, and enrollment in clinical trials as understanding of the pathogen and resultant disease evolve.

Our findings support the potential for the ISI as an aid for early risk stratification of patients presenting to the ED with respiratory complaints early in a pandemic caused by a novel respiratory virus. Importantly, we developed the ISI using a model developed on an unrelated cohort of patients with signs or suspicion of infection that predated the COVID-19 pandemic [17], illustrating its versatility in the risk stratification of infection by a variety of pathogens. In the current study, red band patients experienced a 3-day mortality of 10.9%, nearly 10-times higher than that of green band patients. Furthermore, the mortality of red band patients remained higher at 7-days in both the total population and amongst those with SARS-CoV-2 infection (Fig 2, Table 1). These findings suggest that ED clinicians should evaluate this population with urgency and consideration for movement to a high level of care. While ED clinicians admitted 93.5% of red band patients, including 39.1% to the ICU (Fig 3D and 3E), they also expressed concern for deterioration of patients in the green band, admitting 52.7% in total, including 13.2% to the ICU. The median length of stay for these low-risk patients was comparatively short, as indicated by the high number hospital-free days. In appropriate situations, green band patients may benefit from admission to low-acuity units or outpatient monitoring [13] with close follow-up. Since the numbers of green band patients admitted (n = 96) was higher than the number of red (n = 46) and yellow (n = 43) band patients admitted combined, it appears that the test has the potential to conserve a meaningful amount of physical and personnel resources.

In addition to risk stratification for mortality and hospitalization, the ISI reflects the patients’ clinical courses with respect to complex, well-validated severity of illness scores. Because clinical indicators of organ dysfunction lag behind the underlying biologic process [40–42], we assessed the ISI compared to the maximum SOFA score over the first three days of hospitalization as well as APACHE II scores. The ISI results within minutes of presentation, and it was consistent with scoring systems (Fig 3A and 3C) that require cumulative laboratory and physiologic data captured over several hours to days. Finally, the ISI also identifies a population of patients who may exhibit improvement within the first 3 hospital days (Fig 3B), which could facilitate case management and discharge planning [43].

Importantly, the ISI risk stratifies patients with SARS-CoV-2 infection with the same degree of precision as other patients included in the study. As an assay for the host response, the ISI is a means of quantifying the state of immune activation. Since dysregulated immunity leads to organ dysfunction in severely affected patients, any pathogen causing dysregulated immune response will yield an elevated score [17]. Although we conducted this study primarily in the respiratory care unit of an ED in a high-prevalence area for SARS-CoV-2, as the study was performed prior to the availability of rapid and routine COVID-19 testing, the majority of patients enrolled (66.0%) were either not tested for SARS-CoV-2 or tested negative. This finding is reflective of the uncertainty in practicing Emergency Medicine during a pandemic caused by a novel, poorly-understood pathogen. Nonetheless, among patients testing positive for the novel pathogen, the ISI risk stratification was consistent with SOFA and APACHE II scores and reflected the greater need of high-risk patients for hospitalization and intensive care (Fig 4) independent of the pathogen detection. These findings imply that the ISI can assist ED clinicians in the risk stratification and direction of care for patients before the results of diagnostic tests are available. Furthermore, because the ISI has a rapid turnaround time, it can assist ED clinicians in allocating diagnostics, reserving the more rapid and often scarce tests for those in whom rapid pathogen detection is most critical.

The ISI is a measure of innate immune activity [15–17]. As a result, processes that lead to morbidity or mortality by alternative mechanisms may not result in higher scores. This is apparent in the two green band patients who died in the first three days–one experienced out-of-hospital cardiac arrest and the other a drug overdose. The ISI result was consistent with the fact that neither death was caused by immune dysfunction. Also, patients who present early in the course of infection, prior to the development of immune dysfunction, may be classified into the green band. In these cases, repeated testing may indicate developing immune dysregulation that was not evident initially. Finally, because the ISI quantifies structural changes of leukocytes, conditions known to impact leukocyte structure (e.g., hematologic malignancies, cytotoxic chemotherapy) may impact ISI results.

Our study has several limitations. Though the patient population is diverse and the enrollment pragmatic, it is unclear if the findings of this exploratory single-center study will generalize to other institutions or patient populations. Also, our study was conducted early in the pandemic when infection control concerns limited research staff, so we used remnant blood from patients who had a CBC performed for standard of care for ISI measurement; thus, we were unable to uniformly assess the ISI against commonly measured biomarkers. The study population has a significant representation of black and white patients but does not have significant representation of Hispanic or Asian patients. Because enrollment required a CBC, some degree of risk stratification by the attending providers existed prior to enrollment. Additionally, this study evaluates a single ISI measurement early in the ED course, so we are unable to assess how the ISI trends with progression or resolution of illness. Finally, at the time of enrollment, there was no standardized treatment of COVID-19, so the outcomes may be more representative of the natural course of the disease rather than the current state of treatment.

## Conclusion

The ISI is a pathogen-agnostic, quantitative measure of immune activation; in patients with suspected infection, elevated scores indicate a state of immune dysregulation and portend an immediate risk of adverse outcomes while lower scores portend a less-severe course. As evidenced by its performance during the early COVID-19 pandemic, the ISI may improve efficiency in resource allocation by offering rapid pre-diagnostic risk stratification of patients with suspected infection, independent of the pathogen. This finding is especially important in times of increased ED volumes, prolonged wait times, and diagnostic test delays that are found in a pandemic caused by a novel and evolving pathogen such as SARS-CoV-2. As our understanding of the pathogen and resultant illness evolves, the ISI may help stratify patients for potential interventions. In less than ten minutes and using 100 microliters of whole blood, the ISI has the potential to provide reliable prognostic information that currently requires hours or days to accumulate. In the future, the ISI may provide useful diagnostic and prognostic information for patients with suspected infection and possible immune dysregulation that can lead to life-threatening organ dysfunction.

## Supporting information

S1 Checklist CONSORT 2010 checklist of information to include when reporting a randomised trial*. (PDF)

S1 File Additional figure on Trends in the need for life support (advanced oxygen-delivery and vasopressor support) across ISI interpretation bands for SARS-CoV-2 positive subjects. SOFA (Sequential [Sepsis-Related] Organ Failure Assessment) Score Calculation Procedure.
(DOCX)

S1 Dataset Spreadsheet including all data pertaining to the clinical characteristics, ISIs, and interpretation bands per subject. (CSV)
