# Supplementary material for: Assessment of a cellular host response test to risk-stratify suspected COVID-19 patients in the Emergency Department setting
Source: PLoS One. 2022 Mar 16;17(3):e0264220. doi: 10.1371/journal.pone.0264220 (PMC8926179; doi:10.1371/journal.pone.0264220)
Supplement: S1 File — SOFA (Sequential [Sepsis-Related] Organ Failure Assessment) Score Calculation Procedure. (DOCX) [file pone.0264220.s002.docx]

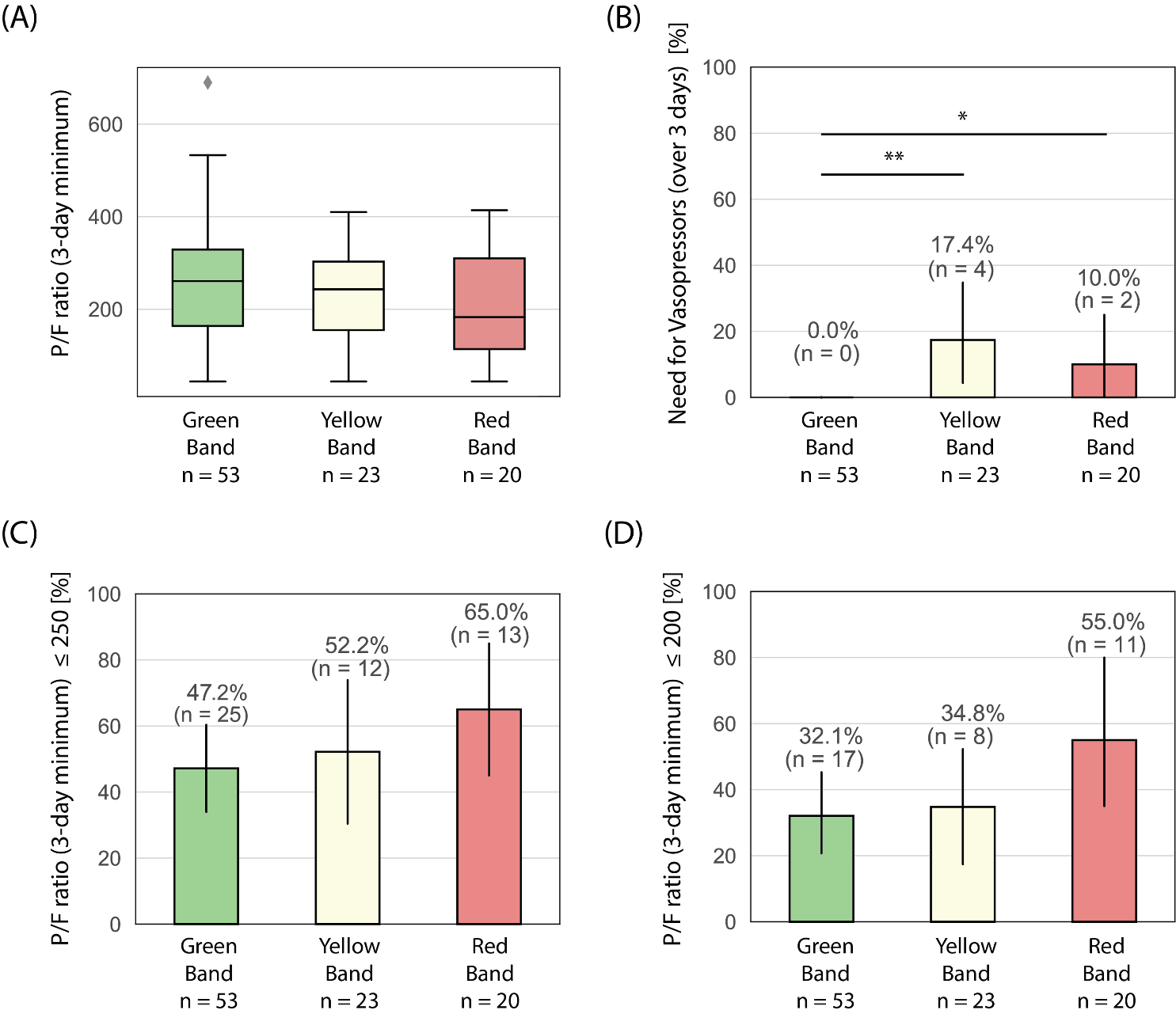


SI-Fig. 1: Trends in the need for life support (advanced oxygen-delivery and vasopressor support) across ISI interpretation bands for SARS-CoV-2 positive subjects: (A) Minimum PaO2 / FiO2 ratio over up to 3-days, (B) need for vasopressors over up to 3-days; (C) minimum PaO2 / FiO2 ratio of less than 250 over up to 3-days; (D) minimum PaO2 / FiO2 ratio of less than 200 over up to 3-days.

Box plots: lines in the boxes, medians; the box ends, interquartile ranges (IQR); whiskers, 1.5x IQR; diamonds, outliers. Bar graphs: Bars, percentages; error pars, 95% confidence intervals. p-values were obtained from an unpaired two-sample Welch’s t-test, with the null hypothesis that the mean of the two samples are equal. p-values reported as * p < 0.05, ** p < 0.01, and *** p < 0.001.

.

**SOFA (Sequential [Sepsis-Related] Organ Failure Assessment) Score Calculation Procedure**

SOFA calculation requires obtaining the values in following table. The SOFA score in this study will be calculated at baseline, Day 1 (Day E), Day 2 and Day 3. Baseline SOFA is calculated using the best value (the value that would lead to the lowest score) within 6 months of enrollment date.

When no baseline data are available in the medical record for any of the elements of SOFA, record as a score of 0 (e.g., assume normal values). Recorded values should be the worst value (defined as those values that would result in the highest score) during specific calendar days (defined as 00:00:00 through 23:59:59) for Day 1, Day 2 and Day 3.

In situations where the patient is in the hospital but there are no data available for individual values, record the subject’s previous value. For example, if a patient has a bilirubin recorded as 1.4 mg/dL on Day 1, no value for Day 2, and 0.9 mg/dL for day 3, then day 2 should be recorded as 1.4 md/dL.

In situations where patients are discharged prior to day 3, no value should be entered for days that the patient is not in the hospital. Other than baseline, no SOFA calculations are performed on days that patients are not in the hospital.

Wildly aberrant or errant values should be disregarded as such and should not be used in calculations.

SOFA Value Table^a^

|  | Value | | | | |
| --- | --- | --- | --- | --- | --- |
|  | 0 | 1 | 2 | 3 | 4 |
| Respiratory  PaO_2_ ^1^/ FiO_2_^2^, mmHg (kPa) | ≥400 (53.3) | <400 (53.3) | <300 (40) | <200 (26.7) with respiratory support (on ventilator) | <100 (13.3) with respiratory support (on ventilator) |
| Coagulation  Platelets,  (x10^3^/µL) | ≥150 | <150 | <100 | <50 | <20 |
| Cardiovascular^3^ | MAP ≥ 70 mmHg | MAP < 70 mmHg | Dopamine ≤5 or  PE <100  Any dose of:  Vasopressin  Milrinone  Dobutamine^b^ | Dopa 5.1-15 or  Epi ≤0.1 or Norepi ≤0.1^b^ or PE 100-300 | Dopa >15 or  Epi >0.1 or  Norepi >0.1^b^ or  PE >300 |
| Central Nervous System  Glasgow Coma Scale score^c^ (GCS) | 15 | 13 – 14 | 10 – 12 | 6 – 9 | <6 |
| Renal  Creatinine,mg/dL (µmol/L) or Urine Output (UOP), mL/d(cc/day) | <1.2 (110) | 1.2 – 1.9 (110-170) | 2.0 – 3.4 (171-299) | 3.5 – 4.9 (300-440)  Or  UOP <500 | > 5.0 (440)  Or  UOP <200 |
| Liver  Bilirubin,  mg/dL  (µmol/L) | < 1.2  (20) | 1.2 – 1.9  (20-32) | 2.0 – 5.9  (33-101) | 6.0 – 11.9  (102-203) | ≥12  (204) |

^a^ 2016 Sepsis-3 consensus standard SOFA Score

^b^ Catecholamine doses are given as μg/kg/min for at least 1 hour.

^C^ Glasgow Coma Scale scores range from 3-15; higher score indicates better neurological function.

1. When an arterial blood gas (ABG) is unavailable for direct measurement of the PaO_2_ , use the conversion table below under Respiratory to estimate PaO_2_ from the SPO_2_
2. For patients on supplemental oxygen, please use the following procedures / conversions

- Nasal Cannula: Use the conversion table below under Respiratory
- Face Mask: Use the conversion table below under Respiratory
- Venturi Mask: Use the documented FiO_2_
- Trach Collar: Use the documented FiO_2_
- Vapotherm: See below for estimated FiO_2_
- Ventilator: Use the documented FiO_2_

1. MAP is measured in mmHg. Doses of dopamine (Dopa), epinephrine (Epi), and norepinephrine (Norepi) are in micrograms / kg / min (conversion from micrograms / minute may be required). Phenylephrine(PE) is in micrograms per minute. Vasopressin is in Units / min.

- Vasopressors must be administered for at least one hour at the defined dose.
- In cases where weight conversions are necessary, use the admit weight

*Respiratory:*

In many cases, ABG data are not available for determination of PaO_2_. When these data are not available, use the following tables to estimate PaO_2_ (or pO_2_) from available SPO_2_ data and to estimate FiO_2_ from available device / flow data.

Example:

PaO_2_/FiO_2_ = 112/.28 = 400 (0 respiratory score)

Use can also use the following calculator to calculate PaO_2_/FiO_2_: <http://www.tidalvolumecalculator.com/page/PaO2FIO2-Ratio>

From the medical record:

SPO_2_ or SO_2_ = 98 (then per table below PaO_2_ =112)

Nasal Cannula = 2 (then per table below FiO_2_ = 28% or 0.28)

For high flow nasal cannula devices (such as Vapotherm, etc), flow values greater than 10L should use the FiO_2_ that the high-flow device reports (a value between .21 and 1.0). Flow values greater than 6 and less than 10 should maximize to 6L NC no matter the FiO_2_ (i.e., use 44%). If no FiO_2_ is available, use 40%.

| Estimated PaO_2_ from SPO_2_ | | | |
| --- | --- | --- | --- |
| SPO_2_ | PaO_2_ | SPO_2_ | PaO_2_ |
| 80 | 44 | 90 | 60 |
| 81 | 45 | 91 | 62 |
| 82 | 46 | 92 | 65 |
| 83 | 47 | 93 | 69 |
| 84 | 49 | 94 | 73 |
| 85 | 50 | 95 | 79 |
| 86 | 52 | 96 | 86 |
| 87 | 53 | 97 | 96 |
| 88 | 55 | 98 | 112 |
| 89 | 57 | 99 | 145 |

<http://intensive.org/epic2/Documents/Estimation%20of%20PO2%20and%20FiO2.pdf>

| Method | O_2_ Flow (LPM) | Estimated FiO_2_ (%) |
| --- | --- | --- |
| Nasal Cannula | 1 | 24 |
|  | 2 | 28 |
|  | 3 | 32 |
|  | 4 | 36 |
|  | 5 | 40 |
|  | 6 | 44 |
| Face Mask | 5 | 40 |
|  | 6-7 | 50 |
|  | 7-8 | 60 |
| Nonrebreather Mask | 15 | 100 |

<http://intensive.org/epic2/Documents/Estimation%20of%20PO2%20and%20FiO2.pdf>

*Coagulation / Platelets:*

Platelet values can be found in the medical record lab values under complete blood counts (CBC).

Values which the laboratory reports as ‘clumped’ should be disregarded; in these cases, record the repeated value, if available. If there are no data available for the hospitalization in question, record 0 or if available, record the subject’s baseline value (best value in the last 6 months prior to ED encounter). If more than one platelet value is collected for Day 1, Day 2 or Day 3, use the worst value.

*Cardiovascular:*

When direct measurements of the Mean Arterial Pressure (MAP) are not available (for example, from an arterial line), use the following formula to calculate MAP from Non-invasive Systolic Blood Pressure (SBP) and Diastolic Blood Pressure (DBP) measurements:

MAP = 1/3 (SBP – DBP) + DBP

If multiple blood pressures are documented for each day, take the worst MAP from concomitant systolic and diastolic blood pressure.

You can also use the following MAP calculator (to calculate MAP from SBP and DBP): <https://www.mdcalc.com/mean-arterial-pressure-map>

*Central Nervous System GCS:*

In cases where bedside staff record the Glasgow Coma Scale (GCS), use the recorded value. If GCS values are not available, the physician’s physical exam and nursing assessments may be used to calculate the GCS based on the following parameters (see Attachment 2):

| Summary of the GCS Rating | | | | | |
| --- | --- | --- | --- | --- | --- |
| Eyes Opening | | Verbal Response | | Best Motor Response | |
| 4 | Spontaneous | 5 | Oriented | 6 | Obeys commands |
| 3 | To sound | 4 | Confused | 5 | Localizing |
| 2 | To pressure | 3 | Words | 4 | Normal flexion |
| 1 | None | 2 | Sounds | 3 | Abnormal flexion |
| NT | Non testable | 1 | None | 2 | Extension |
|  |  | NT | Non testable | 1 | None |
|  |  |  |  | NT | Non testable |

https://www.glasgowcomascale.org/

In situations where values are unable to be ascertained (for example, a patient who is chemically sedated or intubated and Not Testable), record the GCS as 15, giving a 0 SOFA point for that specific instance.

*Creatinine / Urine Output:*

If no value is recorded in the medical record for creatinine within the comprehensive metabolic panel labs, use the most recently available value from the same hospitalization. If there is no value from the pertinent hospitalization, record the value as a 0 point, or the patient’s baseline value, if available (best value in the last 6 months prior to ED encounter). If more than one creatinine value is collected on Day 1, Day 2, or Day 3, use the worst value. For urine output, when the period in question does not involve a complete 24 hour period, use hourly rates for urine output as follows:

<500 cc/day = <21 cc/hour

<200 cc/day = <9 cc/hour

If a patient has End-Stage Renal Disease (ESRD), record a 0 score for each SOFA assessment.

*Bilirubin:*

If no value is recorded in the medical record within the comprehensive metabolic panel labs, use the most recent value (from the same hospitalization). If there is no value from the current hospitalization, enter a 0 point or the patient’s baseline, if available (best value in the last 6 months prior to ED encounter). If more than one bilirubin value is collected on Day 1, Day 2, or Day 3, use the worst value.
